# Correction: Nevirapine hair and plasma concentrations and HIV-1 viral suppression among HIV infected ante-partum and post-partum women attended in a mother and child prevention program in Maputo city, Mozambique

**DOI:** 10.1371/journal.pone.0281190

**Published:** 2023-01-26

**Authors:** The PLOS ONE Staff

## Notice of republication

This article was republished on December 29, 2022, to correct for errors in the Data Availability statement. The publisher apologizes for the errors. Please download this article again to view the correct version. The originally published, uncorrected article and the republished, corrected article are provided here for reference.

## Supporting information

S1 File Originally published, uncorrected article. (PDF)

S2 File Republished, corrected article. (PDF)
