# Supplementary material for: Correction: Nevirapine hair and plasma concentrations and HIV-1 viral suppression among HIV infected ante-partum and post-partum women attended in a mother and child prevention program in Maputo city, Mozambique
Source: PLoS One. 2023 Jan 26;18(1):e0281190. doi: 10.1371/journal.pone.0281190 (PMC9879520; doi:10.1371/journal.pone.0281190)
Supplement: S1 File — (PDF) [file pone.0281190.s001.pdf]

Check for updates

OPEN ACCESS

Citation: Cuco RM, Loquiha O, Juga A, Couto A, Meggi B, Vubil A, et al. (2022) Nevirapine hair and plasma concentrations and HIV-1 viral suppression among HIV infected ante-partum and post-partum women attended in a mother and child prevention program in Maputo city, Mozambique. PLoS ONE 17(2): e0261522. https://doi.org/10.1371/journal.pone.0261522

Editor: Douglas F. Nixon, Cornell University Joan and Sanford I Weill Medical College, UNITED STATES

Received: April 4, 2021

Accepted: December 5, 2021

Published: February 10, 2022

Copyright: © 2022 Cuco et al. This is an open access article distributed under the terms of the Creative Commons Attribution License, which permits unrestricted use, distribution, and reproduction in any medium, provided the original author and source are credited.

Data Availability Statement: ****NOTE TO PA/E AT ACCEPT: please follow up with authors for repository info and URLs/DOIs**** Data will be held in a public repository such as URLs and DOIs after acceptance for publication.

Funding: RMC was the only author who benefitted of an award for doctoral degree at Gent University. This work was funded by VRIL-UOS Flemish

RESEARCH ARTICLE

Nevirapine hair and plasma concentrations and HIV-1 viral suppression among HIV infected ante-partum and post-partum women attended in a mother and child prevention program in Maputo city, Mozambique

Rosa Marlene Cuco[1,2,3]*, Osvaldo Loquiha[4], Adelino Juga[4], Aleny Couto[2], Bindiya Meggi[5], Adolfo Vubil[5], Esperança Sevene[1], Nafissa Osman[1,6], Marleen Temermam[3,7], Olivier Degomme[3], Mohsin Sidat[1], Nilesh Bhatt[5]

1 Faculty of Medicine, Eduardo Mondlane University, Maputo, Mozambique, **2** National Directorate of Public Health, Ministry of Health, Maputo, Mozambique, **3** International Centre for Reproductive Health (ICRH), University of Gent, Gent, Belgium, **4** Department of Mathematics and Informatics, Faculty of Sciences Eduardo Mondlane University, Maputo, Mozambique, **5** Instituto Nacional de Saúde (INS), Marracuene, Mozambique, **6** Department of Gynecology and Obstetrics at Maputo Central Hospital, Maputo, Mozambique, **7** Department of Gynecology and Obstetrics, Aga Khan University Hospital, Nairobi, Kenya

* marlene.cuco@gmail.com

Abstract

Introduction

Prevention of mother to child transmission of HIV (PMTCT) is frequently challenged by irregular access to more effective anti-retroviral therapy. Nevirapine single dose (sdNVP), sdNVP+AZT+3TC for MTCT prophylaxis and NVP+ AZT+3TC for treatment and PMTCT were withdrawn due to low genetic resistance barrier and low efficacy. However current PMTCT lines in Mozambique include DTG+3TC+TDF, TDF+3TC+EFV, DTG +ABC+3TC, and AZT + NVP syrup prophylaxis for exposed babies. We assessed NVP hair and plasma concentrations and association with HIV-1RNA suppression among HIV+ ante-partum and post-partum women under PMTCT in Maputo, Mozambique.

Methods

From December 2013 to November 2014, prospectively were enrolled 200 HIV+ ante-partum women on 200mg nevirapine and zidovudine 300 plus lamivudine 150mg twice daily at least with 3 months treatment and seen again at 24 weeks post-partum. Self-reported pill-taking adherence, NVP concentrations in hair, plasma, hemoglobin, CD4 cell count, HIV-1 RNA load was evaluated. NVP concentration in hair and plasma was analyzed as categorical quartile variable based on better data fit. NVP concentration was set between ≤3.77 ng/ml in plasma and ≤17,20 ng/mg in hair in quartile one to ≥5.36 ng/ml in plasma and ≥53.21 ng/mg in hair in quartile four. Logistic regression models for repeated measures were

Interuniversity Council Development Cooperation, Belgium and by Eduardo Mondlane University, Mozambique. The funders had no role in the study, data collection, analysis, decision to publish or preparation of the manuscript.

Competing interests: The authors have declared that no competing interests exist.

calculated. Following the World Health Organization (WHO) guidelines we set viral suppression at HIV-1RNA < 1000 c/mL. Outcome was HIV-1 RNA<1000 copies/ml. Predictor was NVP concentration in hair categorized in quartiles.

Results

In total 369 person-visits (median of 1.85) were recorded. Self-reported adherence was 98% (IQR 97–100%) at ante-partum. In 25% person visits, NVP concentrations were within therapeutic levels (3.77 ng/ml to 5.35 ng/ml) in plasma and (17.20 ng/mg to 53.20 ng/mg) in hair. In 50% person visits NVP concentrations were above 5.36 ng/ml in plasm and 53.21 ng/mg in hair. HIV-1 RNA suppression was found in 34.7% of women with two viral loads, one at enrollment and another in post-partum. Odds of HIV-1 RNA suppression in quartile 4, was about 6 times higher than in quartile 1 (p-value = 0.006) for NVP hair concentration and 7 times for NVP plasma concentration (p-value = 0.012).

Conclusions

The study results alert for potential low efficacy of current PMTCT drug regimens in use in Mozambique. Affordable means for individual monitoring adherence, ART plasma and hair levels, drug resistant and HIV-1 RNA levels monitoring are recommended for prompt identification of inadequate drug regimens exposure patterns and adjust accordingly.

Introduction

Prevention of mother to child transmission of HIV (PMTCT) is frequently challenged by irregular accessibility to more effective anti-retroviral therapy (ART) [1, 2] and limited routine monitoring of HIV-1 RNA [3, 4] particularly in HIV/AIDS most affected and low resourced sub-Saharan African countries like Mozambique [5, 6] Timely identification of HIV-1 viral unsuppressed ante-partum and post-partum women and implementation of suitable measures is key for prevention of MTCT of HIV infection [7].

The HIV prevalence in Mozambique is one of the highest, including among ante-natal attendees with an estimated of 15.8% in 2019 [8] Nevertheless there are over 90% of HIV positive pregnant women under ART for PMTCT in the country, but appropriate HIV-1RNA load monitoring was available to less than 50% [9] and HIV-1 RNA suppression was estimated at 45% at 12 months post-partum with decreasing tendency thereafter [10]. Similarly, the rate of mother to child transmission of HIV (MTCT) increased from 11% in 2014 to 14% in 2019 [11, 12]. Pregnant and post-partum women should continuously access effective ART and routine monitoring of HIV-1 RNA load to prevent reduce MTCT of HIV infection [7].

For more than 15 years, nevirapine (NVP) was a backbone non-nucleoside reverse transcriptase inhibitor (NNRTI) of the World Health Organization (WHO) recommended line for PMTCT and ART [13]. In Mozambique, NVP was firstly introduced in 2002 as intra partum single dose (Sd NVP) for PMTCT [14–17] and progressively discontinued [13, 15, 16, 18] until more effective combined ART with or without NVP are available [16].

In 2011, based on WHO guidance for PMTCT outcomes improvement [2, 18] Mozambique moved from starting ART at 28 weeks to 14 weeks of pregnancy or sooner, with NVP either Sd NVP intra-partum combined to AZT + 3TC for mothers, option A, or NVP as a composite of

maternal triple-drug provided throughout ante-partum and breastfeeding the option B, as well as NVP or AZT syrup for exposed infants until 4–6 weeks post-partum [18].

Since 2012 to curtail unsatisfactory PMTCT outcomes associated to NVP based ART [19–21] first line regimens containing efavirenz (EFV), tenofovir (TDF), and dolutegravir (DTG) with two nucleoside reverse transcriptase inhibitors (NRTI's) were successively introduced [22] However, until 2019 low access and affordability led to the continued use of NVP, particularly in form of AZT300+3TC150+NVP200 prescribed twice daily for adults as alternative to AZT + 3TC+ EFV and TDF+3TC+EFV [16, 22] HIV exposed infants continued receiving AZT +NVP syrup and for treatment they were medicated with AZT+3TC+NVP (3FDC baby), abacavir (ABC)+3TC+NVP (2FDC+NVP50). Only 17% of infants were receiving new pediatric formulations recommended by WHO with DTG+3TC +TDF [11, 23] Only in 2020 the main first line DTG+3TC+TDF and the alternatives TDF+3TC+EFV, TDF+3TC+ABC were available countrywide, prophylaxis for exposed babies still AZT+NVP syrup [22, 24].

Adequate treatment for HIV positive mothers to achieve HIV1 RNA suppression is key for attaining 2030 UNAIDS PMTCT targets [9] Nevertheless, similar to other settings, in Mozambique, monitoring of ART exposure and early detection of HIV1-RNA unsuppressed women on MTCT program is still a challenge [1, 4]. Therapeutic drug monitoring (TDM) is the gold standard that shows the drug concentrations in plasm [3, 4, 25–27] But its usefulness is limited to some hours to few days and there is wide intra-individual variability [25]. Additionally, the TDM in plasma is relatively expensive [28] as it requires specialized technology for sampling and testing, hampering routine use in low-income countries [25–27].

In contrast, the evaluation of ART concentration in hair is relatively affordable [2, 3]. Results have shown hair concentration to be a strong independent predictor of HIV-1 RNA load suppression [4, 27, 29]. The ART concentrations in hair reflects trends of historic medication taken by a patient over a long period from days up to months [29–33].

This study aimed to assess self-reported adherence, measurements of NVP in hair and plasma and effects on HIV viral suppression among ante-partum and post-partum women under treatment with NVP+ AZT+ 3TC provided at routine PMTCT in three urban primary health centers in Maputo city, Mozambique. We hypothesized that ante-partum and post-partum women would have low self-reported adherence, and as consequence a low NVP levels in hair and plasma and low HIV-1RNA suppression. We also considered that NVP levels would enable predictions of HIV-1RNA suppression.

Material and methods

An observational prospective study was conducted from December 2013 to November 2014 in three primary public health facilities (Primeiro de Maio Health Center, Primeiro de Junho Health Center and Mavalane Health Center) located in the Mavalane health area in Maputo City, Mozambique. The three health facilities attend 60% of ante-partum and post-partum women on ART with estimated HIV prevalence among ante-partum women of 24% in 2018 [23]. In 2019, 90.000 HIV positive women became pregnant more than 60% were under TDF + 3TC+ EFV and 10% of ante-partum and post-partum women were still on NVP based ART regimens. The remaining were under DTG +3TC+ ABC and other alternative ART lines.

Sample size

Conveniently and prospectively, we selected 200 HIV positive ante-partum women from the three ANC/PMTCT services at the above-mentioned healthcare facilities. Ante-partum women who met the selections criteria were enrolled after they provided informed consent.

All HIV positive ante-partum women aged 18 years or older on at least three months on ART with AZT+3TC+NVP regardless of the gestational age period and willing to be seen again at 24 weeks post-partum were eligible for the study. All women arriving for antenatal visit were invited to participate in the study and consenting women were enrolled consecutively until sample size being achieved for the study. All women were seen at two time points: ante-partum and six months after delivery.

A structured questionnaire was used to collect socio-demographic and self-reported adherence data. We used four days adherence tool recommended by the AIDS Clinical Trials Group (ACTG) to assess ART regimen and number of pills taken modified by the authors to include plasm and hair NVP concentrations levels [34] Hair and blood samples were collected to measure NVP concentrations in both study time periods in enrolled women.

Hair sample collection and measurement of nevirapine concentration

Approximately 10 to 20 strands (around 1–3 mg) of hair were obtained from occipital region from each woman [27, 29]. The hair samples were individually labeled and assembled in plastic bags and stored at the room temperature at the health facility before being sent to the National Institute of Health (INS) laboratory located in Maputo city, Mozambique. Hair samples were sent for analysis to the laboratory at the University of California San Francisco (UCSF) in United States of America (USA). The therapeutic drug monitoring (TDM) was performed by using liquid chromatography/tandem mass spectrometry (LC/MS/MS) [3, 27, 29, 35]. In brief, NVP was extracted in methanol/trifluoroacetic acid in a proportion of 9:1 and shaken at 37˚C in a water bath overnight and submitted to liquid-liquid extraction in alkaline conditions [29, 35]. The lower limit of quantification of NVP was 0.5 ng/mg (LLOQ). NVP hair therapeutic levels are ranged from 3.77 ng/mg to 5.35 ng/mg.

Blood sampling and measurement of nevirapine concentration

Five ml of whole blood was collected in EDTA tubes to measure NVP concentration in woman during ante-partum and at post-partum period. The collected blood samples were centrifuged at 3,000 rpm for 3 minutes within six hours after collection at the INS laboratory. Plasma samples were stored in cryovials at the -20˚C until final shipment to the University of Stellenbosch in South Africa for analysis. NVP concentration in plasma were measured through validated high performance liquid chromatography (HPLC) [36] NVP lower limit of quantification (LLOQ) was 25 ng/ml. NVP blood therapeutic levels was set between 3,000–8,000 ng/mL.

HIV-1 viral load, CD4+ T cells and full blood count

An additional 5ml of the whole blood sample was collected in EDTA tube and sent to INS laboratory within 6 hours after collection for HIV-1 RNA viral load using COBAS Amp li Prep /COBAS Taq Man HBV test, v2.0 (Roche Diagnostics, Germany), a fully-automated system that employs real-time PCR technology with a limit of detection of ≤50 copies/ml. T-lymphocyte (CD4 cells) quantification was performed by using FACS Caliber flow cytometer (Becton Dickinson, USA) using the Multistep software (Becton Dickinson, USA) within 24 hours after sample collection at INS laboratory. Full blood count including three-part differential of hematological parameters was performed within 6 hours of blood collection in Vacutainer® tubes with K2 (EDTA) anticoagulant (Becton Dickinson, USA) using the hematology analyzer Sysmex KX21N (Sysmex Corporation, Japan).

Statistical analysis

The primary outcome was HIV-1 RNA load<1000 copies/mL following the World Health Organization (WHO) guidelines [13, 15]. And primary predictors were NVP levels in hair and plasma at each study visit. Summary statistics were computed for a set of baseline characteristics. A log-transformed viral load was calculated to compare the viral loads between visits using a Wilcoxon signed-rank test. Mixed-effects logistic regression models for repeated measures were used to estimate and compare the association of NVP concentration levels in hair, plasma and HIV1-RNA. Following previous model [27]. NVP levels in hair and plasma were analyzed as categorical variable and quartiles. The best estimation of categorization was based on data fit and NVP therapeutic levels in hair and plasma. The model was adjusted by age groups and number of visits. We were unable to adjust for time of ART initiation due to unclear dates. All analyses were performed using SAS/STAT software, version 9.4.

Ethical Approval: Mozambican National Bioethics Committee approved the study protocol (IRB 357/CNBS/12). The Ministry of Health, Maputo City Directorate also provided administrative clearance. Written and oral informed consent was obtained from each enrolled woman.

Results

Socio-demographic characteristics of pregnant and post-partum women

The "Table 1" presents the demographic characteristics of the study participants, as well as NVP concentration in hair and plasma for the 200 ante- and post-partum women enrolled in this study. All women were under AZT+3TC+NVP as part of their own health and PMTCT. Each patient contributed on average with 1.85 visits to the analysis (range: 1–2) for a total of 369 person-visits.

Of the 200 women, 86.5% were younger than 35 years, 27.5% had either secondary or higher education and 10.5% were employed at the time of study roll out. Median hemoglobin was 10.5 g/dl (IQR: 9.6–11.4), median CD4 count 377.5 cells/μl (IQR:270.3–491.3), median HIV-1 viral load was 170 copies/ml (IQR: 29.0–4233.0).

As shown in "Table 2" in 44% of person visits women had viral load counts equal or above 1000 copies/ml. NVP concentration varied from $\leq$ 3.77 ng/ml in plasm and $\leq$ 17,20 ng/mg in hair in quartile1, to $\geq$ 6.98 ng/ml in plasma and $\geq$ 106.89 ng/mg in hair in quartile 4. However 25% of women in quartile 1 had NVP levels below therapeutic threshold of ($\leq$ 3.77 ng/ml) in plasm and ($\leq$ 17.20 ng/ml) in hair.

In approximately 25% and 50% of person visits, women had NVP concentration in hair and in plasma within and higher therapeutic levels respectively.

"Fig 1" displays the percent of person-visits in each quartiles of NVP concentration level in hair and plasma related to HIV suppression (HIV-1 viral load <1,000 copies/ml). There was a strong association between NVP concentration levels in the hair with HIV-1 viral load < 1,000 copies/mL (p <0.01) than NVP concentration levels in the plasma (p = 0.030).

Virology outcome at enrollment (ante-partum) and at post-partum

Of the 200 women, only 49 (24.5%) had their HIV-1 RNA results available for the two visits. All women with and without HIV viral suppression were identical in terms of age, education level, HIV-1 RNA load and CD4+ cells count. Virology suppression was found in 17 (34.7%) of women, among them 11 (22.4%) had HIV-1 RNA <1000cp also at enrolment and 6 (12.2%) had discordant results with HIV-1 RNA > 1000cp at ante-partum and < 1000 cp at postpartum. Among the failure group, the majority (22 or 44%) had HIV-1 RNA <1000cp at ante-partum and HIV-1 RNA > 1000cp at post-partum and 10 (20.4%) had HIV-1 RNA > 1000cp at

Table 1. Baseline patient characteristics (N = 200).

	n	Percent
**Age (years)**		
**15–24**	55	27.5%
**25–34**	118	59.0%
**≥35**	21	10.5%
**Missing**	6	3.0%
**Median age (IQR)**	28 years (24–32)	
**Education level**		
**None**	5	2.5%
**Primary**	57	28.5%
**Secondary/High**	55	27.5%
**Missing**	83	41.5%
**Employed**		
**No**	95	47.5%
**Yes**	21	10.5%
**Missing**	84	42.0%
**Baseline Hemoglobin**		
**<8 g/dl**	3	1.5%
**8–12 g/dl**	117	58.5%
**12–16 g/dl**	25	12.5%
**Missing**	55	27.5%
**Median hemoglobin (IQR)**	10.5 g/dl (9.6–11.4)	
**Baseline CD4 count**		
**≤199 cells/μl**	22	11.0%
**200–499 cells/μl**	124	62.0%
**500–749 cells/μl**	46	23.0%
**Missing**	8	4.0%
**Median CD4 count (IQR)**	377.5 cells/μl (270.3–491.3)	
**Baseline viral load**		
**≤49 copies/ml**	47	23.5%
**50–999 copies/ml**	54	27.0%
**≥1000 copies/ml**	46	23.0%
**Missing**	53	26.5%
**Median viral load (IQR)**	170 copies/ml (29.0–4233.0)	

*Note: there were 53 samples that were either not processed or with incomplete information.

https://doi.org/10.1371/journal.pone.0261522.t001

ante-partum and at post-partum. The HIV-1 RNA at post-partum was significantly higher than at enrollment for the virology failure group (p<0.0001), but not on the virology suppressed group (p = 0.093).

Median and interquartile range (IQR) of nevirapine concentration levels in the plasma and hair by viral load count at visit 1 and 2

The median nevirapine concentration levels were slightly high for women with viral load count <1000 cp/ml for both plasma and hair measurements at any visit. The overall median nevirapine concentration in the plasma for women with HIV-1RNA load count <1000 cp/ml was 5ng/ml (IQR: 4–85 ng/ml) and 6 ng/ml (IQR: 2–75 ng/ml) for HIV-1RNA load count >1000 cp/ml was 3ng/ml (0-6ng/ml) ante-partum and 6ng/ml(2-7n/ml) no post-partum. The

Table 2. Viral load and nevirapine concentration quartiles in hair and plasma.

**Viral Load**		
	**n**	**Percent**
Person-visits contributing to study (n = 369, percent[†])		
**Viral load**		
**<1000 copies/mL**	122	58.1%
**≥1000 copies/mL**	88	41.9%
**NVP hair concentration levels (quartiles)**		
**Quartile 1 (≤ 17.20)**	71	24.8%
**Quartile 2 (17.21–53.20)**	72	25.2%
**Quartile 3 (53.21–106.88)**	72	25.2%
**Quartile 4 (≥106.89)**	71	24.8%
**NVP blood concentration levels (quartiles)**		
**Quartile 1 (≤ 3.77)**	42	25.1%
**Quartile 2 (3.78–5.35)**	42	25.1%
**Quartile 3 (5.36–6.97)**	43	25.7%
**Quartile 4 (≥6.98)**	40	24.0%

† percent excluding missing values in each person-visit

IQR: Interquartile range.

https://doi.org/10.1371/journal.pone.0261522.t002

overall median nevirapine concentration in the hair for women with HIV-1RNA load count <1000 cp/ml in hair was (59 ng/mg (IQR: 23–109 ng/mg) and 19 ng/mg (IQR: 0–73 ng/mg for HIV-1RNA load >1000 cp/ml was (3ng/mg (0-63ng/mg) ante-partum and (19ng/mg(0-75ng) in post-partum. The"Fig 2A" shows Nevirapine plasma and hair concentration levels by HIV-1RNA load at ante-partum, awhile "Fig 2B" shows Nevirapine plasma and hair concentration levels by HIV-1RNA load at post-partum.

The median concentration levels differed significantly between HIV-1RNA load count groups for hair measurements at post-partum visit (p<0.001) "Table 3".

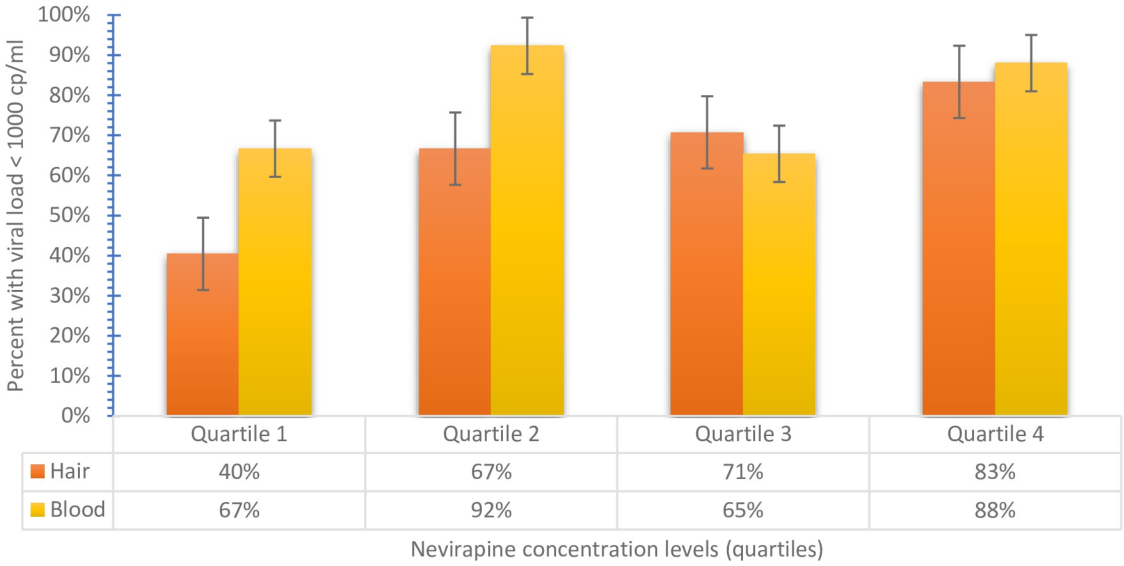

Fig 1. Displays the percent of person-visits in each quartiles of NVP concentration level in hair and plasma related to HIV suppression (HIV-1 viral load <1,000 copies/ml).

https://doi.org/10.1371/journal.pone.0261522.g001

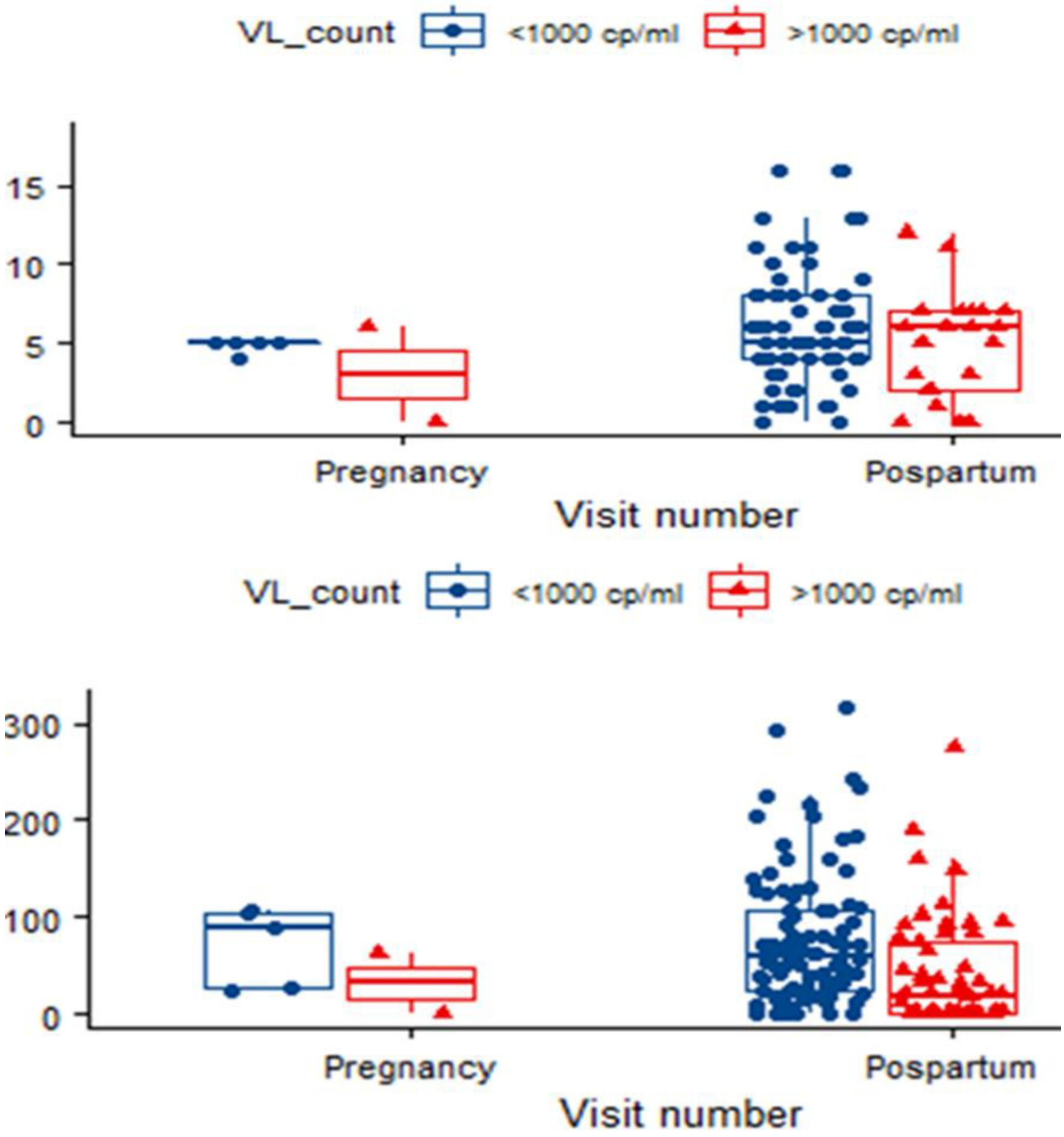

Fig 2. a. shows Nevirapine plasma and hair concentration levels by HIV-1RNA load at ante-partum, awhile. b shows Nevirapine plasma and hair concentration levels by HIV-1RNA load at post-partum.

https://doi.org/10.1371/journal.pone.0261522.g002

Association of NVP in hair and plasma concentration with HIV-1 viral load suppression

"Table 3" presents the results of the mixed-effects model for HIV-1 viral load $< 1,000$ copies/mL, adjusted for age and visit number. We could not adjust for time of ART initiation due to mostly unclear date for ART initiation.

For a random patient, increased NVP concentration levels either in the hair or in plasma were significantly associated with a higher odds ratio for HIV-1RNA load $< 1,000$ copies/ml. For person-visits with NVP hair concentration in quartile 4, the odds of HIV-1 viral

Table 3. Odds ratio (95% confidence interval) for the association of NVP hair and blood concentration with viral load < 1000 copies/mL (for all person-visits).

	Odds ratio (95% CI)	
	**Hair**	**Blood**
**Age groups (years)**		
**15–24**	1.00	1.00
**25–34**	1.21 (0.61–239)	1.21 (0.59–2.46)
**≥35**	1.36 (0.39–4.81)	1.45 (0.39–5.44)
**Visit number**		
**1**	1.00	1.00
**2**	0.27 (0.13–0.59)‡	0.38 (0.17–0.82)‡
**NVP concentration levels**		
**Quartile 1**	1.00	1.00
**Quartile 2**	2.02 (0.86–4.74)	11.28 (2.05–31.93)‡
**Quartile 3**	2.67 (1.11–6.41)‡	1.70 (0.62–4.69)
**Quartile 4**	6.16 (1.73–21.99)‡	7.15 (1.57–32.60) ‡

‡ p-value <0.05

AIC = 263.2, for regression model with NVP hair concentration levels

AIC = 257.3, for regression model with NVP blood concentration levels.

https://doi.org/10.1371/journal.pone.0261522.t003

load < 1,000 copies/ml were about 6 times higher than that of NVP hair concentration in quartile 1 (p-value = 0.006). Similarly, for NVP plasma concentration in quartile 4, the odds of HIV-1RNA load < 1,000 copies/ML was about 7 times higher than that of NVP blood concentration in quartile 1 (p-value = 0.012).

The "Fig 3" also display plots of adjusted odds ratio with 95% confidence interval for the association of NVP hair and blood concentration with HIV-1RNA viral load < 1000 copies/mL (for all person-visits). The odds of HIV-1RNA load < 1,000 copies/mL was significantly lower for visit number two when compared with visit number one. Age wasn't a significant factor in the virology outcome.

Discussion

Effective ART exposure is critical for virology suppression [7, 27, 33] and PMTCT. In 2019, DTG+3TC+TDF was introduced and alternative line included TDF+3TC+EFV [24]. The AZT and NVP syrup were still used for prophylaxis among HIV exposed children [24]. In the same year the PMTCT coverage in Mozambique was over 90% among HIV positive pregnant women. However, HIV-1RNA suppression was 45%, MTCT was 14%, a very high rate when compared to the neighbouring countries [8, 37–39] Maternal HIV-1RNA load at delivery is a high predictor of MTCT of HIV infection [40, 41]. The low rates of HIV1-RNA suppression and high MTCT are suggestive of the need of affordable HIV-1RNA /TDM monitoring and DRM testing for personalized clinic decisions particularly for childbearing women.

We carried out a TDM and HIV-1RNA load trends. We hypothesized, ante-partum and post-partum women would have low self-reported adherence, low NVP concentrations in plasma and in hair, low HIV-1RNA suppression with consequent increased risk for MTCT of HIV infection. Differently to our hypothesis we found a short term high self-reported adherence, but 25% of women have low NVP level in plasma and in hair, these findings support a long term low adherence at least among these women as hair concentration gives a long period up to months of drug intake behaviour. Also, among other women we cannot roll out the

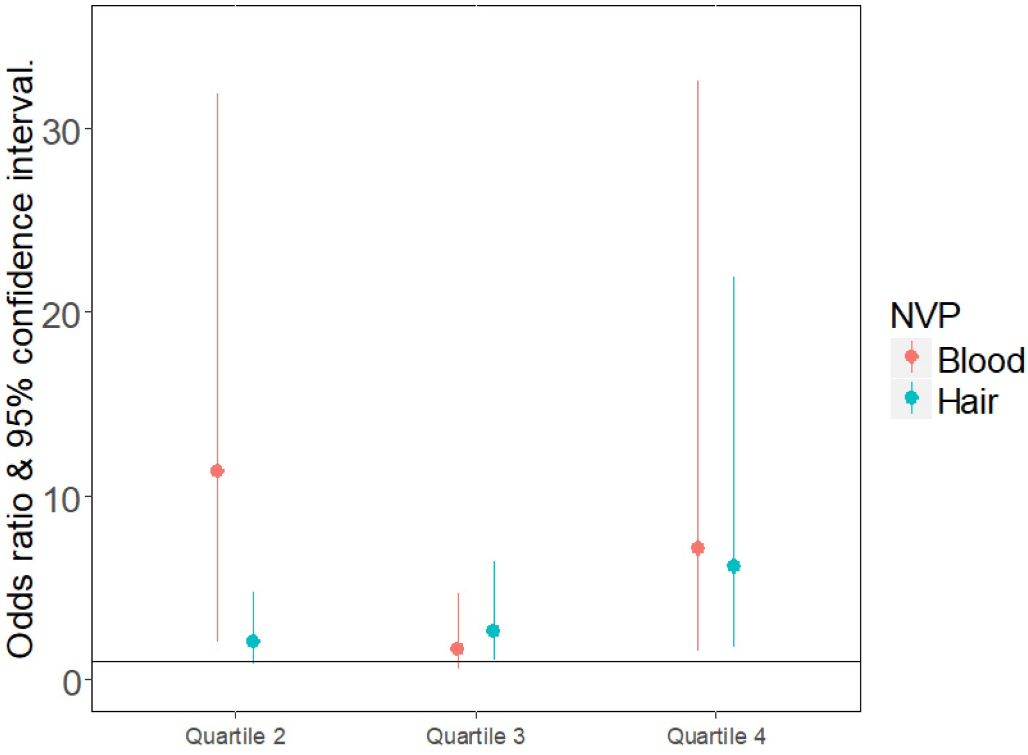

Fig 3. Also display plots of adjusted odds ratio with 95% confidence interval for the association of NVP hair and blood concentration with HIV-1RNA viral load < 1000 copies/mL (for all person-visits).

https://doi.org/10.1371/journal.pone.0261522.g003

possibility of instable adherence throughout ante and post- partum period particularly, worsening at post-partum. The possibility of resistant mutations also is present However50% person visits NVP levels were above 5.36 ng/ml in plasm and 53.21 ng/mg in hair and in 25% person-visits NVP concentration were within therapeutic ranges, but HIV-1RNA suppression occurred only in 17 (34.7%) of women with two measurements. Consequently, failing to show adequate NVP hair concentration as an independent predictor of HIV-1 RNA suppression. Perhaps, the failure to demonstrate our predictions may be due to small sample size and convenient sampling. Nevertheless, our findings were consistent in demonstrating an increase in NVP plasma and hair levels positivity in random patients associated with increased odds of HIV-1 RNA suppression. The odds of HIV-1 RNA suppression in quartile four of NVP levels in hair was over six-time higher than in quartile 1; 6.16 (95% 1.73–21.99; p-value = 0.006) [23, 29, 43]. Although the odds of HIV-1 RNA suppression of NVP in plasma was higher, 7.15 (1.57–32.60; p-value = 0.012), the confidence interval was wider, favouring comparatively weaker association. Also, it is important to acknowledge that NVP plasma concentration levels provide very short information of drug availability in the blood stream, from several minutes to some days and is prone to the bias of time gap between drug uptake and blood withdraw [29, 42] Whereas hair concentration represents a lengthier period of drug exposure in the most body fluids [25, 27]

Furthermore, among the failure group, the majority (22 or 66.6%) of women had HIV-1 RNA at post-partum significantly higher than at enrolment (p<0.0001) and at any visit the median nevirapine levels were slightly low in the failure group (HIV-1RNA count >1000 cp/ml) for both plasma (6 ng/ml (IQR: 2–75 ng/ml) and hair (19 ng/mg (IQR: 0–73 ng/mg) and median NVP concentration levels differed significantly between HIV-1RNA load count groups

only for hair measurements at postpartum visit ($p<0.001$). Earlier identification of HIV-1RNA and therapeutic status would have enabled early detection of HIV-1RNA failure in at least 66.6% women and better guided drug switch on 31% to recover from probable HIV-1RNA failure and therefore, reducing the risk of MTCT of HIV infection.

Technical constraints limited the possibilities for assessing other current PMTCT ART components such as EFV, AZT and 3TC, TDF levels including the possible impact of NVP/EFV pharmacogenetics on HIV-1RNA drug exposure [43, 44]. However, we acknowledge HIV-1RNA low suppression, may be also related to AZT monotherapy intake in (sdNVP +AZT+3TC) PMTCT line and consequently HIV-1 resistant mutations selection. Additionally pre-ART drug-resistance to multiple antiretroviral classes like NVP +AZT+3TC is reported to increase the risk of virological failure particularly to EFV [45]. Also low HIV-1RNA failure has been associated to NVP/EFV perhaps due to HIV-1 cross resistance mutations and also related to AZT/3TC and 3TC [45] Additionally, intermittent and discontinued exposure related to sdNVP+AZT+3TC intake has been appointed as important contributor to HIV-1RNA low suppression particularly among HIV + women with high natality rates and short pregnancy spacing as is the case for most Mozambicans of childbearing age [46, 47].

Recent surveys among pregnant women in south rural Mozambique HIV-1 drug resistant mutation (DRM) was around 10% mainly due to NNNRTI and NRTI including NVP, EFV, 3TC, ABC, TDF [48]. In a survey of general population that included childbearing women as participants in south urban and central rural Mozambique [48] 83% were on tenofovir (TDF)/lamivudine (3TC)/efavirenzes (EFV) and the pre-treatment drug resistance PDR (NNRTI) varied from 16.8% to 31.2% in south urban and central rural setting, respectively and mainly due to EFV/NVP drug resistance, mostly among pre-exposed. Acquired drug resistance (ADR) varied from 8.3% to 15.5% and the majority had NNRTI-NRTI dual resistance. The most common NRTI (ADR) was to 3TC/Emtricitabine and Abacavir [48]. These results support the probability of existence of drug resistance for the most first line drugs currently used for PMTCT reinforcing the need for individual HIV1-RNA and drugs resistance status assessment.

Similarly, it is well documented NVP/EFV pharmacokinetics is influenced by CYP2B6 single nucleotide polymorphisms (SNPs) [43, 44] Populations of African origins have the highest genetic diversity of CYP2B6 mostly causing NVP/EFV low clearance and high plasmatic and hair exposure [44]. A Mozambican study found the frequency of CYP2B6 SNPs (516T/785G of (34.7% and 42.6%) and 516T associated to high NVP toxicity among HIV/AIDS patients [48, 49]. Likewise, carriers of CYP2B6 516G>T SNPs under NVP based ART had significantly higher NVP plasma concentration particularly the homozygotes CYP2B6 c.516T/T [41–44, 50] Additionally, over threefold increase on EFV exposure in hair (long) and plasma (short) was reported among African American women [27, 51] Nigerian patients [52] carrier of CYP2B6 516TT genotype. However, the role of CYP2B6 NPSs on low viral suppression still not clear [27].

The CYP2B6 NPSs rates in Mozambique and others countries support the likelihood of influence of CYP2B6 SNPs on NVP/EFV metabolism and HIV-1RNA response among our study population [27, 43, 52]. Nevertheless, this hypothesis needs further investigations.

The study had some limitations that included a small sample size and thus may not reflect ART behaviours and virology outcomes of all pregnant and postpartum women across the country as a whole. The convenient sampling of study sites and selection of women, the issue of lost to follow-up (LTFU), and some missing information also impacted negatively on study results and do not allow appropriate extrapolations for a larger population of the country.

Conclusion

With the results of this study, we can conclude that for a random participant NVP in hair NVP predicted HIV-1RNA suppression. High NVP levels in hair and plasma, and low HIV-1 RNA suppression, such as what was documented in this study likely suggests emerging HIV-1 RNA NVP resistant mutations and virology and therapeutic failure and possible effect of CYP2B6 NPSs. These findings alert for potential low efficacy of current and future NVP/EFV based ART treatment that are still being used in childbearing women and infants in Mozambique. Though cannot be generalized in overall results reinforce the need of individual HIV-1RNA monitoring, DRM and pharmacogenetic testing for prompt identification of erratic drug exposure patterns and adjust accordingly, to improve NVP/EFV based ART outcomes and prevent MTCT of HIV infection.

A larger study to determine the magnitude of hair NVP exposure, DRM, virology outcomes at PMTCT settings is needed because NVP/EFV, 3TC and AZT based regimes are still in use throughout the country. Whenever possible, HIV-1RNA, load measurement, assessment of DRM at ante-partum and post-partum period could be helpful to identify women at risk and adequate measures are taken to minimize MTCT of HIV infection. Pregnant women unique identifier number could uplift PMTCT efforts and improved follow-up.

Supporting information

S1 Data.
(XLSX)

Acknowledgments

The University of California Sao Francisco (UCSF) and Dr. Monica Gandhi team for their technical support that allowed us to benefit from advanced laboratory technology to determine NVP in hair. We are also grateful to all women for participating in this study. Authors also would like to thank the valuable support of health staff.

Author Contributions

Conceptualization: Rosa Marlene Cuco, Osvaldo Loquiha, Bindiya Meggi, Adolfo Vubil, Nafissa Osman, Marleen Temermam, Olivier Degomme, Mohsin Sidat, Nilesh Bhatt.

Data curation: Rosa Marlene Cuco, Osvaldo Loquiha, Adelino Juga, Olivier Degomme, Mohsin Sidat, Nilesh Bhatt.

Formal analysis: Rosa Marlene Cuco, Osvaldo Loquiha, Adelino Juga, Aleny Couto, Bindiya Meggi, Adolfo Vubil, Marleen Temermam, Olivier Degomme, Mohsin Sidat, Nilesh Bhatt.

Funding acquisition: Marleen Temermam.

Investigation: Rosa Marlene Cuco, Bindiya Meggi, Mohsin Sidat.

Methodology: Rosa Marlene Cuco, Osvaldo Loquiha, Aleny Couto, Adolfo Vubil, Esperança Sevene, Marleen Temermam, Olivier Degomme, Mohsin Sidat, Nilesh Bhatt.

Project administration: Rosa Marlene Cuco.

Resources: Marleen Temermam.

Supervision: Esperança Sevene, Nafissa Osman, Marleen Temermam, Olivier Degomme, Mohsin Sidat.

Validation: Mohsin Sidat, Nilesh Bhatt.

Writing – original draft: Rosa Marlene Cuco, Osvaldo Loquiha, Adelino Juga, Bindiya Meggi.

Writing – review & editing: Esperança Sevene, Nafissa Osman, Marleen Temermam, Olivier Degomme, Mohsin Sidat, Nilesh Bhatt.

References

1. Kusumaningrum A, Ibrahim F, Yunihastuti E, Bela B. HIV Drug Resistance after Failure of 6 Month First-line Therapy in a Hospital: A Case Series. Acta Med Indones. 2019 Jul; 51(3):253–2571. PMID: 31699949
2. WHO Ua U. TOWARDS UNIVERSAL ACCESS Scaling up priority HIV/AIDS interventions in the health sector Progress Report 2010. 2010. Access at https://www.who.int/hiv/pub/2009progressreport/e
3. Hickey DM, Salmen RC, Tessler RA, Omollo D, Bacchetti P, Magerenge R, et al. Antiretroviral concentrations in small hair samples as a feasible marker of adherence in rural Kenya J Acquir Immune Defic Syndr 2014; 66(3):311–5. https://doi.org/10.1097/QAI.0000000000000154 PMID: 24694932
4. Koss CA, Natureeba P, Mwesigwa J, et al. Hair concentrations of antiretrovirals predict viral suppression in HIV-infected pregnant and breastfeeding Ugandan women [published correction appears in AIDS. 2015 Nov; 29(17):2369]. AIDS. 2015; 29(7):825–830. https://doi.org/10.1097/QAD.0000000000000619 PMID: 25985404
5. Sebunya R, Musiime V, Kitaka SB, Ndeezi G. Incidence and risk factors for first line anti-retroviral treatment failure among Ugandan children attending an urban HIV clinic. AIDS Res Ther. 2013; 10(1):25. Published 2013 Nov 11. https://doi.org/10.1186/1742-6405-10-25 PMID: 24215971
6. Micek MA, Dross S, Blanco AJ, et al. Transmission of nevirapine-resistant HIV type 1 via breast milk to infants after single-dose nevirapine in Beira, Mozambique. J Infect Dis. 2014; 210(4):641–645. https://doi.org/10.1093/infdis/jiu130 PMID: 24596282
7. Chetty T, Newell M, Thorne C, Coutsoudis A. Viraemia before, during and after pregnancy in HIV-infected women on antiretroviral therapy in rural KwaZulu-Natal, South Africa, 2010–2015. Tropical Medicine and International Health 2018. https://doi.org/10.1111/tmi.13001 PMID: 29121445
8. UNAIDS: Global AIDS Update 2018 | MSF SAMU. Access at https://samumsf.org/. . ./hiv/hiv-response/unaids-global-aids-update-20189
9. UNAIDS. Joint United Nations Programme on HIV/AIDS. Fast-Strategy to End HIV/AIDS Epidemic in 2030. 2018. https://www.unaids.org/en/resources/campaigns/World-AIDS-Day-Report-2014
10. INS INdS, INE INdE, GTM GTMdCaHS. Ronda de Vigilância Epidemiológica do HIV e sífilis em Mulheres Grávidas em Moçambique,: Principais Resultados; Maputo, Moçambique. 2011. Access at https://www.misau.gov.mz/index.php/relatorios-anuais. Portal sobre saúde em Moçambique MISAU PNC ITS HIV/SIDA Relatórios Anuais Instituições do Governo Portal do Governo Assembleia da República Presidência da República.
11. Ministério da Saúde. Relatório anual das atividades de prevenção da transmissão vertical 2019. Access at https://www.misau.gov.mz/index.php/relatorios-anuais. Portal sobre saúde em Moçambique MISAU PNC ITS HIV/SIDA Relatórios Anuais Instituições do Governo Portal do Governo Assembleia da República Presidência da República.
12. Joint United Nations Program on HIV/AIDS (UNAIDS). UNAIDS Spectrum—National HIV Estimates 2019. Geneva, Switzerland: Joint United Nations Program on HIV/AIDS (UNAIDS), 2019.
13. WHO. GUIDANCE ON GLOBAL SCALE-UP OF THE PREVENTION OF MOTHER-TO-CHILD TRANSMISSION OF HIV Towards universal access for women, infants and young children and eliminating HIV and AIDS among children. World Health Organization 2007.Access at https://www.who.int/hiv/mtct/PMTCT_enWEBNov26.pdf
14. Ministry H. Mozambique, Relatório Preliminar Avaliação Nacional do Programa PTV 2011 Access at https://www.misau.gov.mz/index.php/relatorios-anuais. Portal sobre saúde em Moçambique MISAU PNC ITS HIV/SIDA Relatórios Anuais Instituições do Governo Portal do Governo Assembleia da República Presidência da República.
15. Eshleman SH, Jackson JB. Nevirapine resistance after single dose prophylaxis. AIDS reviews. 2002; 4 (2):59–63. Epub 2002/08/03. PMID: 12152519.
16. Ministério da Saúde. Plano Nacional de Eliminação da Transmissão Vertical, Moçambique 2012–2015. 2012. Access at https://www.misau.gov.mz/index.php/relatorios-anuais. Portal sobre saúde em Moçambique MISAU PNC ITS HIV/SIDA Relatórios Anuais Instituições do Governo Portal do Governo Assembleia da República Presidência da República.

17. Giaquinto C, Rampon O, Rossi A. Antiretroviral therapy for prevention of mother-to-child HIV transmission: focus on single-dose nevirapine Clinical Drug Investigation 2006; 26(11): 16. https://doi.org/10.2165/00044011-200626110-00001 PMID: 17163296
18. Nachega JB, Hislop M, Dowdy DW, Gallant JE, Chaisson RE, Regensberg L, et al. Efavirenz versus nevirapine-based initial treatment of HIV infection: clinical and virological outcomes in Southern African adults. AIDS 2008; 22(16):8. https://doi.org/10.1097/QAD.0b013e328310407e PMID: 18832875
19. Lockman S, Hughes MD, McIntyre J, Zheng Y, Chipato T, Conradie F, et al. Antiretroviral therapies in women after single-dose nevirapine exposure Randomized Controlled Trial. N Engl J Med. 2010; 363 (16). https://doi.org/10.1056/NEJMoa0906626 PMID: 20942666
20. UNAIDS, PEPFAR, UNICEF, WHO. Global Plan 2016n on the fast-track to an AIDSfree generation the incredible journey of the global plan towards the elimination of new HIV infections among children by 2015 and keeping their mothers alive 2016. 2016.https://old.aidsdatahub.org/sites/default/files/publication/UNAIDS
21. WHO. World Health Organization. Programmatic update: Use of antiretroviral drugs for treating pregnant women and preventing HIV infection in infants. Update on antiretroviral regimens for treating and preventing HIV infection and update on early infant diagnosis of HIV: interim guidance Geneva: World Health Organization; 2018 (WHO/CDS/HIV/1819) Licence: CC BY-NC-SA 30 IGO. 2012.
22. Ministério da Saúde. Mozambique, Normas Prevenção da Transmissão Vertical 2011. Access at https://www.misau.gov.mz/index.php/relatorios-anuais. Portal sobre saúde em Moçambique MISAU PNC ITS HIV/SIDA Relatórios Anuais Instituições do Governo Portal do Governo Assembleia da República Presidência da República.
23. Ministry of Health Mozambique file Epidemiological Surveillance Round of HIV and Syphilis in Mozambique, 2011 3 Results Caractérizacão of participants in the round Of the total of 14,449 data sheets of pregnant women in Mozambique. Access at https://www.misau.gov.mz/index.php/inqueritos-de-saude
24. Ministério da Saúde. Normas clínicas atualizadas para Seguimento de Paciente HIV Positivo. 2019. Access at https://www.misau.gov.mz/index.php/relatorios-anuais. Portal sobre saúde em Moçambique MISAU PNC ITS HIV/SIDA Relatórios Anuais Instituições do Governo Portal do Governo Assembleia da República Presidência da República.
25. Nettles RE, Kieffer TL, Parsons T, Johnson J, Cofrancesco JJ, Gallant JE, et al. Marked intraindividual variability in antiretroviral concentrations may limit the utility of therapeutic drug monitoring. Clin Infect Dis 2006; 15(42):1189–96. https://doi.org/10.1086/501458 PMID: 16575741
26. Punyawudho B, Singkham N, Thammajaruk N, Dalodom T, Kerr SJ, (2016) Therapeutic drug monitoring of antiretroviral drugs in HIV-infected patients, Expert Review of Clinical Pharmacology, 9:12, 1583–1595, https://doi.org/10.1080/17512433.2016.1235972 PMID: 27626677
27. Baxi SM, Greenblatt RM, Bacchetti P, Jin C, French LA, Keller JM, et al. Nevirapine Concentration in Hair Samples Isa Strong Predictor of Virologic Suppression in a Prospective Cohort of HIV-InfectedPatients2015. 10(6): e0129100.
28. Haberer J, E., Kiwanuka J, Nansera D, Muzoora C, et al. Real-time Adherence Monitoring of Antiretroviral Therapy Among HIV-infected Adults and Children in Rural Uganda. AIDS 2013; 27(13). https://doi.org/10.1097/QAD.0b013e328363b53f PMID: 23751260
29. Huang Y, Gandhi M, Greenblatt RM, Gee W, et al. Sensitive analysis of anti-HIV drugs, efavirenz, lopinavir and ritonavir, in human hair by liquid chromatography coupled with tandem mass spectrometry. NIH Public Access. 2008; 22(21):3401–9. https://doi.org/10.1002/rcm.3750 PMID: 18837069
30. Beumer JH, Bosman IJ, Maes RA. Hair as a biological specimen for therapeutic drug monitoring. Int J Clin Pract. 2001; 55(6):353–7. J Acquir Immune Defic Syndr. 2014 Jul 1; 66(3): 311–315. https://doi.org/10.1097/QAI.0000000000000154 PMID: 11501221
31. Gandhi M, Ameli N, Bacchetti P, Anastos K, et al. Atazanavir Concentration in Hair Is the Strongest Predictor of Outcomes on Antiretroviral Therapy. Clinical Infectious Diseases 2011; 52(10):1267–75. https://doi.org/10.1093/cid/cir131 PMID: 21507924
32. Duval X, Peytavin G, Breton G, Ecobichon JL, at al. Hair versus plasma concentrations as indicator of indinavir exposure in HIV-1-infected patients treated with indinavir/ritonavir combination. AIDS. 2007 Jan 2; 21(1):106–8. https://doi.org/10.1097/QAD.0b013e3280118486 PMID: 17148976
33. Gandhi M, Ameli N, Bacchettic P, Ganged SJ, et al. Protease Inhibitor Levels in Hair Samples Strongly Predict Virologic Responses to HIV Treatment. AIDS. 2009; 23(4):471–8. https://doi.org/10.1097/QAD.0b013e328325a4a9 PMID: 19165084
34. Fletcher CV, Testa MA, Brundage RC, Chesney M, et al. Four Measures of Antiretroviral Medication Adherence and Virologic Response in AIDS Clinical Trials Group Study 359. J Acquir Immune Defic Syndr 2005; 40(3):301–6 (ISSN: 1525-4135). https://doi.org/10.1097/01.qai.0000180078.53321.6a PMID: 16249704

35. Olds KP, Kiwanuka PJ, Nansera D, Huang Y, et al. Assessment of HIV antiretroviral therapy adherence by measuring drug concentrations in hair among children in rural UgandaAIDS Care. 2015; 27(3):327–32. https://doi.org/10.1080/09540121.2014.983452 PMID: 25483955

36. Srivastava P, Moorthy SG, Gross R, Barrett SJ. A Sensitive and Selective Liquid Chromatography/Tandem Mass Spectrometry Method for QuantitativeAnalysis of Efavirenz in Human Plasma PLoS ONE 2013; 8(6). https://doi.org/10.1371/journal.pone.0063305

37. Chetty T NM, Thorne C, Coutsoudis A. Viraemia before, during and after pregnancy in HIV-infected women on antiretroviral therapy in rural KwaZulu-Natal Souht Africa, 2010–2015. Tropical Medicine and International Health. 2018; 23:79–91. https://doi.org/10.1111/tmi.13001 PMID: 29121445

38. Ministério da Saúde. Inquérito de Indicadores de Imunização, Malária e HIV/SIDA em Moçambique (IMASIDA). Caverton, Maryland, USA: MISAU, INE e ICFI 2015. https://dhsprogram.com/pubs/pdf/SR246/SR246.pdf

39. WHO. World Health Organization.Mother-to-child transmission of HIV -WHO 2018. https://www.who.int/hiv/topics/mtct/en/

40. WHO. World Health Organization. Prevention of mother-to-child transmission (PMTCT). Global Health Observatory (GHO) data2018. https://www.who.int/gho/hiv/epidemic_response/PMTCT_text/en/m Prevention of mother-to-child transmission (PMTCT)

41. Mock PA, Shaffer N, Bhadrakom C, Siriwasin W, at al. Maternal viral load and timing of mother-to-child HIV transmission, Bangkok, Thailand. B[53]angkok Collaborative Perinatal HIV Transmission Study Group. AIDS. 1999; 13(3):7. Epub Epub 1999/04/13.

42. Arvold ND, Ngo-Giang-Huong N, McIntosh K, Suraseranivong V, at al. Maternal HIV-1 DNA load and mother-to-child transmission. AIDS Patient Care STDS. 2007; 21(9):5. Epub 2007/10/09. https://doi.org/10.1089/apc.2006.0169

43. Gandhi M, Devi S, Bacchetti P, Chandy S, Heylen E, Phung N, et al. Measuring Adherence to Antiretroviral Therapy via Hair Concentrations in India. J Acquir Immune Defic Syndr. 2019. https://doi.org/10.1097/QAI.0000000000001993 PMID: 30865182

44. Mhandire D., Lacerda M., Castel S., Ku Mhandire K., Zhou D., Swart M., et al. Effects of CYP2B6 and CYP1A2 Genetic Variation on Nevirapine Plasma Concentration and Pharmacodynamics as Measured by CD4 Cell Count in Zimbabwean HIV-Infected. OMICS A Journal of Integrative Biology. 19(9). https://doi.org/10.1089/omi.2015.0104 PMID: 26348712

45. Maseng MJ, Tawe 1L, Thami PK, Seatla KK, Moyo S, Axel Martinelli A, et al. Association of CYP2B6 Genetic Variation with Efavirenz and Nevirapine Drug Resistance in HIV-1 Patients from Botswana. Pharmacogenomics and Personalized Medicine. 2021. https://doi.org/10.2147/PGPM.S289471 PMID: 33758532

46. Miln RS SL, Beck IA, Mckrnan-Mullin J, Deng W, Sibley TR, Dross S, et al. Minority and majority pre-treatment HIV-1 drug resistance associated with failure of 1st-line NNRTI ART in Kenyan women. HEALTH EDUCATION RESEARCH Theory & Practice AIDS. 2020.

47. Ruperez M, Noguera-Julian M, Gonzalez R, Maculuve S, Bellido R, Vala A, et al. HIV drug resistance patterns in pregnant women using next generation sequence in Mozambique. PLOSONE| 2018; 13(5). PLOSONE| https://doi.org/10.1371/journal.pone.0196451 PMID: 29742132

48. V. Carnimeo V TI, Fuentes S, Vaz D. D, Schramm B, at qall. High level of HIV drug resistance informs dolutegravir roll-out and optimized NRTI backbone strategy in MozambiqueJAC Antimicrob Resistance 2020. https://doi.org/10.1093/jacamr/dlab050 PMID: 34223118

49. Ciccacci C DFD, Marazzi MC, Rufini S, Mancinelli S, Buonomo E, Giardina E, et al. Association between CYP2B6 polymorphisms and nevirapine-induced SJS/TEN. International Journal of Molecular Sciences. 2013; 69(11):7. https://doi.org/10.1007/s00228-013-1549-x PMID: 23774940

50. Calcagno A DAA, Cusato J, Baietto L, Di Perri G, Rostagno R, Siccardi M, et al. Influence of CYP2B6 and ABCB1 SNPs on nevirapine plasma concentrations in Burundese HIV-positive patients using dried sample spot devices British Journal of Clinical Pharmacology. 2015; 74(1):6. https://doi.org/10.1111/j.1365-2125.2012.04163

51. Gandhi M GR, Peter Bacchetti P, Chengshi J, Huang Y, Anastos K, Cohen M JJ, et al. A Single-Nucleotide Polymorphism in CYP2B6Leads to >3-Fold Increases in Efavirenz Concentrations in Plasma and Hair Among HIV-Infected Women Journal of Infectiuos Disease. 2012; 2012(206):8. https://doi.org/10.1093/infdis/jis508 PMID: 22927450

52. Nwogua JN GM, Owend A, H. Khood SH, Taiwoe B, Olagunjud A, Berzinse B, et al. Associations between efavirenz concentrations, pharmacogenetics and neurocognitive performance in people living with HIV in Nigeria. AIDS. 2021; 35(1):8. https://doi.org/10.1097/QAD.0000000000002984 PMID: 34115651
